# Early and delayed long-term transcriptional changes and short-term transient responses during cold acclimation in olive leaves

**DOI:** 10.1093/dnares/dsu033

**Published:** 2014-10-16

**Authors:** María de la O Leyva-Pérez, Antonio Valverde-Corredor, Raquel Valderrama, Jaime Jiménez-Ruiz, Antonio Muñoz-Merida, Oswaldo Trelles, Juan Bautista Barroso, Jesús Mercado-Blanco, Francisco Luque

**Affiliations:** 1 Departamento de Biología Experimental, Universidad de Jaén, Jaén, Spain; 2 Departamento de Protección de Cultivos, Institute for Sustainable Agriculture (CSIC), Córdoba, Spain; 3 Departamento de Arquitectura de Computadores, Universidad de Málaga, Málaga, Spain

**Keywords:** *Olea europaea*, olive, cold-stress, transcriptomics

## Abstract

Low temperature severely affects plant growth and development. To overcome this constraint, several plant species from regions having a cool season have evolved an adaptive response, called cold acclimation. We have studied this response in olive tree (*Olea europaea* L.) cv. Picual. Biochemical stress markers and cold-stress symptoms were detected after the first 24 h as sagging leaves. After 5 days, the plants were found to have completely recovered. Control and cold-stressed plants were sequenced by Illumina HiSeq 1000 paired-end technique. We also assembled a new olive transcriptome comprising 157,799 unigenes and found 6,309 unigenes differentially expressed in response to cold. Three types of response that led to cold acclimation were found: short-term transient response, early long-term response, and late long-term response. These subsets of unigenes were related to different biological processes. Early responses involved many cold-stress-responsive genes coding for, among many other things, C-repeat binding factor transcription factors, fatty acid desaturases, wax synthesis, and oligosaccharide metabolism. After long-term exposure to cold, a large proportion of gene down-regulation was found, including photosynthesis and plant growth genes. Up-regulated genes after long-term cold exposure were related to organelle fusion, nucleus organization, and DNA integration, including retrotransposons.

## 1. Introduction

Olive (*Olea europaea* L.) is an evergreen species that is sensitive to winter chilling temperatures with severe leaf damage at −7°C.^1^ Olive oil is the major product extracted from olive fruit, and its consumption is steadily growing worldwide. The process of cold acclimation is very complex and involves diverse physiological, metabolic, and developmental changes that are under strict genetic control.^2^ The identity of the cold-stress sensor in plants is yet unknown.^3^ Nevertheless, early changes in membrane rigidification leading to certain cytoskeletal rearrangement has been proposed to be a cellular sensor for cold stress,^4,5^ in addition to metabolic or redox status alterations. Changes in membrane fluidity lead to a transient induction of Ca^2+^ channels and a raised cytosolic Ca^2+^ level, which acts as a second messenger in the early sensing of low temperatures.^6^ Cold acclimation is the result of several processes, such as the synthesis of cryoprotectant molecules and membrane phospholipids, protein stabilization, maintenance of ion homeostasis, and a stress response mainly mediated by the scavenging of reactive oxygen species (ROS).^7–9^ Cold response involves around 1,000 genes in *Arabidopsis*, as previously shown by whole transcriptome analyses.^10,11^ A relevant number of cold-regulated genes encode transcription factors or proteins involved in transcription.^12^ Among these, C-repeat binding factor (CBF)/dehydration responsive element binding consists of transcriptional activators that provide one of the most important pathways for cold response.^13–15^ The expression of the *CBF* genes is induced after an increase of the cytosolic Ca^2+^ level and is responsible for controlling the expression of a large number of cold-response (COR) proteins.^16^ The CBFs bind to the low-temperature-responsive DNA-regulatory element termed C-repeat/dehydration-response element^13,14^ present in the promoters of many COR genes. Nevertheless, the analysis of cold acclimation in the olive drupe and seed has shown an important role of ‘fatty-acid desaturase (FAD)’ genes in cold tolerance and oil quality.^17,18^ These *FAD* genes contribute to cold acclimation by increasing the presence of unsaturated fatty acids which produce cell-membrane fluidification and F-actin stabilization.^19,20^ However, little is known about the gene-expression changes occurring during cold acclimation in this important crop, and no transcriptomic analysis has been conducted to determine the genetic changes induced by cold stress. The aim of this work was to study whole transcriptome changes induced in leaves of olive plants by above-freezing cold stress.

## 2. Materials and methods

### 2.1. Plant material and cold-stress induction

To induce stress by long-term exposure to low temperature, we used 4-month-old potted olive plants of the cv. Picual. These plants were obtained from a commercial nursery located in Córdoba province, southern Spain. Before being subjected to the low-temperature treatment, they were acclimated at 24°C day light for 3 weeks within the same controlled growth chamber where the cold-stress assay was conducted. Afterwards, we incubated a group of 35 plants under the following environmental conditions: 14-h photoperiod of fluorescent light at 65 µmol m^2^ s (10°C day/4°C night) for 10 days and constant 76–78% relative humidity. An additional group of 15 plants remained as a control treatment. Time of cold exposure was considered from the moment at which the growth chamber reached the working temperature for the first time in daylight (i.e. 10°C). Aerial tissues were then harvested at 0 h, 24 h, and 10 days (three plants/time point) after the start of the experiment. Tissue samples were immediately frozen in liquid nitrogen and kept at −80°C until the extraction of total RNA. In order to build an olive transcriptome of diverse biotic and abiotic stresses, mechanical wounding and infection with the soil-borne fungus *Verticillium dahliae* were also considered. This pathogen is the causal agent of Verticillium wilt of olive, one of the most serious diseases affecting olive cultivation in many areas.^21^ Therefore, an additional set of nursery-produced ‘Picual’ plants (40) were submitted to root-dip inoculation in a conidia suspension of a *V. dahliae* isolate representative of the so-called defoliating pathotype, following the procedure detailed in Mercado-Blanco *et al.*^22^ with the modification that inoculation time was only 30 min. Non-inoculated plants (40), but manipulated in the same way (control treatment), were used to obtain tissue samples of wounded plants in the absence of the pathogen. Functional genomics analysis of these responses will be published elsewhere, but the complete ‘olive stress transcriptome’ is referred here for the first time.

### 2.2. RNA sample preparation and high-throughput sequencing

RNA samples were extracted from the leaves of three replicates of control and the 24-h and 10-day cold-stress-treated plants. Total RNA was extracted using a Spectrum Plant Total RNA kit (Sigma-Aldrich, St Louis, MO, USA) according to the manufacturer's instructions. Any DNA contamination was removed by DNase I treatment on column (Roche, Basel, Switzerland). The RNA quality tests were performed with the Agilent 2100 bioanalyser (Agilent Technologies, Santa Clara, CA, USA) using an RNA 6000 Pico assay kit (Agilent Technologies, Santa Clara, CA, USA). We pooled RNA from each time and complementary DNA (cDNA) libraries and sequencing in Illumina HiSeq 1000 sequencer was performed by GeneSystems (Valencia, Spain). Two replicates of each sample were sequenced on different lanes in the flow cell.

### 2.3. Bioinformatic analysis

Quality control of sequencing was performed using NGS_QC software.^23^ After trimming and cleaning, we obtained 149,638,888 paired-end reads of 101 bp length and 35 Phred quality (30.21 Gb). The ABySS-pe 1.3.3 software^24,25^ was used for *de novo* assembly in ‘Picasso’ supercomputer (www.scbi.uma.es). The ABySS software was chosen due to memory use and time limits for users in ‘Picasso’ at that moment, since ABySS had previously shown a good balance between memory use, runtime, and integrity of assembled genes.^26^ The command-line parameters used were abyss-pe k=64 n=10 j=2 name=library1 in=’/library1_R1.fastq library1_R2.fastq’ so that the minimum number of pairs required for building scaffolds was 10. We also included additional read sets corresponding to other stress conditions (Supplementary Table S2). Firstly, each library was assembled separately, producing a number of scaffolds ranging from 96,268 to 276,359. On average, the N50 length, average length, and maximal length for the 8,090,448 scaffolds obtained were 661, 312, and 8,791 bases, respectively. The mapping analysis with bowtie2^27^ showed that 87.4% of reads on average aligned end to end on those scaffolds (bowtie2 parameters: -D 15 -R 2 -N 0 -L 22 -i S,1,1.15) and 93.2% aligned locally (-D 15 -R 2 -N 0 -L 20 -i S,1,0.75). Secondly, to collapse all related sequences that could not have done so during the first assembly step, the scaffolds of all libraries were assembled using ABySS single-end (*k*-mer = 63). Finally, contigs shorter than 200 bases and any sequence with no best hit for a plant but for a different organism were removed from the assembly using BLAST 2.2.20 software^28^ with a minimum expectation value of 1e−5. Mapping analysis with bowtie2 in the same way as above showed that 65% of reads on average aligned end to end on the final unigenes while 83.5% aligned locally. Other quality parameters for the new transcriptome were calculated by means of NGS_QC for the GC percentage, GenoToolBox (https://github.com/aubombarely/GenoToolBox) for basic stats, and Full-LengtherNext 0.0.8 (FLN) for sequence structure analysis.^29^

Sma3s^30,31^ with default parameters was used in ‘Picasso’ supercomputer for functional annotation of all unigenes based on the similarity with UniProt-annotated sequences, in particular, using plant taxonomy. The gene-expression study was made with the DNAStar (ArrayStar 4) Qseq software for RNAseq analysis (www.dnastar.com). We undertook mapping using parameters *k*-mer = 63 and 95% of matches and used the default normalization method of ‘reads per kilobase per million mapped reads', RPKM.^32^ The gene ontology (GO) terms retrieved by Sma3s were loaded as an annotation file or .annot in the Blast2GO suite V2.7.0^33,34^ to carry out the statistical analysis of GO-term enrichment. Blast2GO has integrated the Gossip package for statistical assessment of differences in GO-term abundance between two sets of sequences.^35^ This package employs the Fisher's exact test and corrects for multiple testing. We performed a one-tailed Fisher's exact test. The test was made using a false discovery rate (FDR), with a filter value <0.05. Blast2GO returns the GO terms over-represented at a specified significance value.^34^ The results were saved in a Microsoft Excel datasheet, and charts were generated.

### 2.4. Quantitative real-time–polymerase chain reaction analysis

First-strand cDNA was synthesized from 1 µg of total RNA primed with 60 µM of random hexamer primer and Transcriptor Reverse Transcriptase, using the Transcriptor First Strand cDNA Synthesis Kit (Roche, Basel, Switzerland).

The quantitative real-time polymerase chain reaction (Q-RT–PCR) was performed in a Bio-Rad CFX96 PCR system with master mix SsoFastTM EvaGreen^®^ Supermix (Bio-Rad Laboratories, Hercules, CA, USA), in 10 µl of reaction mixture, containing 10 ng of cDNA. Amplifications were performed under the following conditions: initial polymerase activation at 98°C for 30, then 40 cycles at 98°C for 5 s, and at 60°C for 10 s, followed by a melting step from 65 to 95°C. An internal control of constitutive olive actin was used for the normalization of results. The constitutive normalization control was selected after comparing several genes and primer pairs on different olive tissues and found to be the most constant in expression.^36^ The oligonucleotides used for the amplifications are listed in Supplementary Table S1. Each PCR reaction was performed three times, and three different trees were analysed at every time of cold exposure.

### 2.5. Data availability

The Illumina sequenced read data reported in this article have been deposited in the National Center for Biotechnology Information (NCBI) Sequence Read Archive and are available under the Accession Numbers (NCBI: SRR1525051, SRR1525052, SRR1525231, SRR1525237, SRR1524947, SRR1524948, SRR1524949, SRR1524950, SRR1524951, SRR1524952, SRR1525086, SRR1525087, SRR1525113, SRR1525114, SRR1525213, SRR1525214, SRR1525224, SRR1525226, SRR1525284, SRR1525285, SRR1525286, SRR1525287, SRR1525415, SRR1525416, SRR1525436, SRR1525437). The Transcriptome Shotgun Assembly project has been deposited at DDBJ/EMBL/GenBank under the accession number GBKW00000000. The version described in this article is the first version, GBKW01000000.

## 3. Results and discussion

### 3.1. Environmental conditions and stress symptoms of olive plants under low-temperature treatment

**Figure 1.**
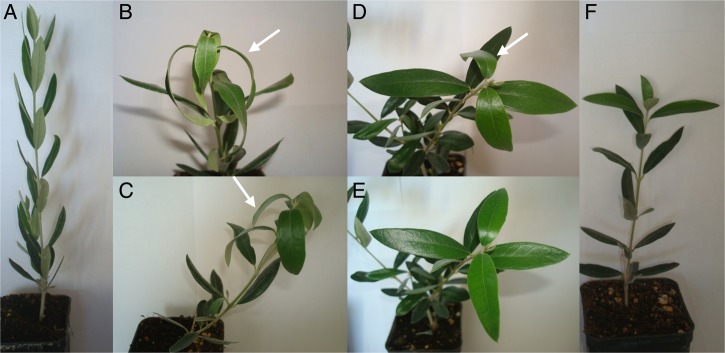
Morphological appearance of ‘Picual’ plants under prolonged cold stress: a 10-day assay*.* Pictures of representative plants subjected to cold stress (10°C day/4°C night, 14-h photoperiod). (A) Control plant; (B) cold-stressed plant during 1 day; (C) cold-stressed plant during 2 days; (D) cold-stressed plant during 3 days; (E) almost fully recovered plant after 6 days of cold stress; (F) fully recovered plant after 10 days of cold stress. The arrows indicate some flaccid leaves.

Olive ‘Picual’ plants subjected to low temperature displayed clearly visible symptoms of stress 24 h after starting the experiment, compared with control plants (Fig. 1A and B). The vast majority of plants had flaccid leaves, resembling wilting symptoms (Fig. 1C and D). However, 4–5 days after the start of the experiment, plants progressively recovered from these symptoms, and nearly no trace of flaccid or wilted leaves remained at the end of the experiment (10 days of exposure to low temperature) (Fig. 1E and F). This result shows that a sudden fall in temperature, even if this is above 0°C, can trigger clear morphological changes and stress symptoms in olive plants. However, in just a few days most plants were fully recovered and were not distinguishable from the unstressed ones. Low-temperature treatment caused an overproduction of H_2_O_2_ accompanied by a down-regulation of SOD isoenzymes, a key enzyme of the ascorbate–glutathione cycle (APX) and an antioxidant NADP-dehydrogenase (NADP-ICDH). During long-term cold acclimation at 10 days, a slight recovery of Mn-SOD and NADP-ICDH was detected, indicating that a mechanism of cold acclimation takes place (Supplementary Fig. S1). This showed the ability of olive plants to rapidly acclimate to prolonged cold stress.

### 3.2. Transcriptome assembly, functional annotation, and expression analysis

**Figure 2.**
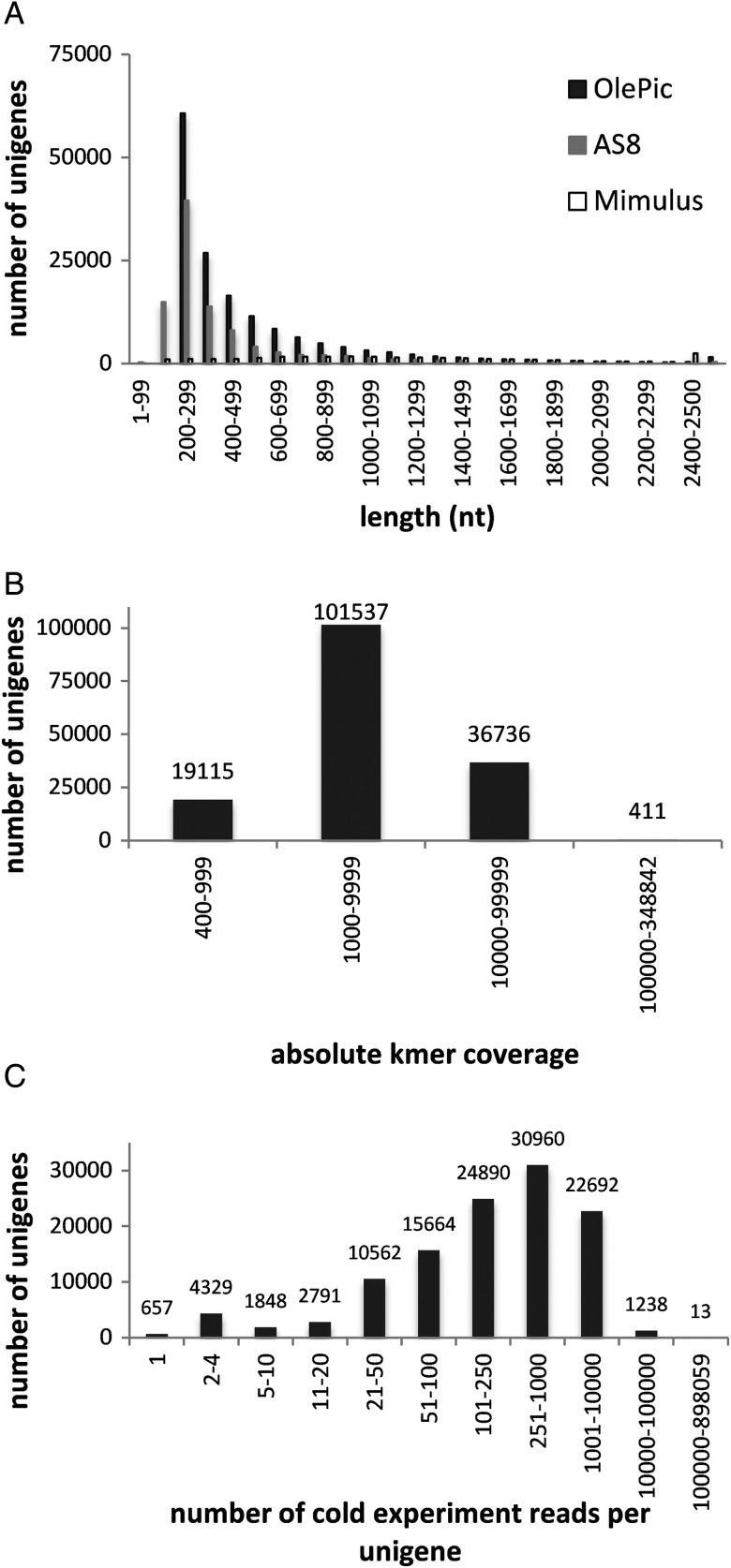
Length distribution from the fully assembled data set and assembly coverage distribution*.* (A) Length (nucleotides) distribution from the fully assembled data set, value labels are shown only for OlePic in a few categories. Value labels are shown only for a few categories. (B) Distribution of *k*-mer coverage for all unigenes originated after the second step of the assembly from all the scaffolds obtained in the first step. (C) End-to-end mapping distribution of the number of cold-stress experiment reads per unigen for all unigenes from the assembled data set.

**Figure 3.**
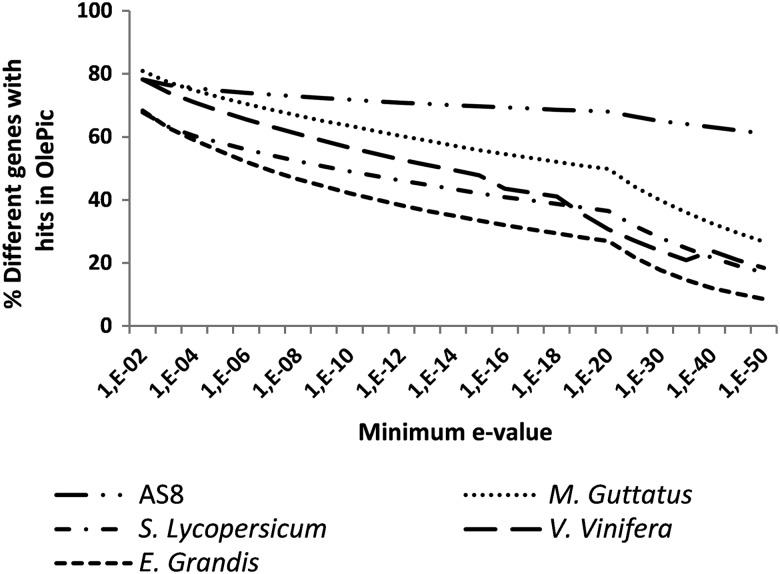
Percentage of different genes that mapped in OlePic as reference at different minimun e-values*.* The cDNA sequences of AS8 and olive-related plant species were mapped to OlePic by using the blastn algorithm from blast/2.2.20 software.

The transcriptome was assembled from a total of 741,674,755 high-quality paired-end reads (149.77 Gb; Supplementary Table S2). Each sample was separately assembled using the ABySS-pe software with a *k*-mer = 64. Finally, all samples were re-assembled together with the ABySS single-end using a *k*-mer = 63. The final transcriptome (OlePic) contains 157,799 unigenes with an N50 length of 700 bases, an average length of 544 bases, a maximal length of 11,114 bases, 46% GC, and 0% undetermined. The distribution by length of the unigenes generated in the assembly is shown in Fig. 2A. The result shows that although OlePic has a higher number of assembled transcripts, the distribution by length is similar to the currently available, most complete olive transcriptome AS8. In addition, OlePic is enriched in longer contigs than AS8 in a similar way as the *Mimulus guttatus* one, the available plant transcriptome most phylogenetically related to Olive tree.^37^ On the other hand, mapping analysis with bowtie2 showed that, on average, 65% of original reads aligned with OlePic end to end and 83.5% aligned locally. It can therefore be concluded that the vast majority of the reads sequenced are represented in this new transcriptome. Another common assessment of the quality of the assembled database is provided by the coverage. The minimum *k*-mer coverage for each unigene was 400 (Fig. 2B), and 88% of contigs were covered for >1,000 *k*-mer of 63 bases. Regarding cold stress, all reads derived from the cold experiment samples were mapped end to end to the assembly (Fig. 2C). In addition, 73.3% of unigenes were covered for reads obtained in this study (115,644 unigenes). From these unigenes, 82.5% were covered by more than at least 50 reads. The OlePic transcripts’ similarity and transcriptome coverage with olive AS8 transcriptome and other phylogenetically related species (monkey flower,^37^ tomato,^38^ grapevine^39^ and rose gum^40^) were measured by blastn algorithm from BLAST software at different expectation values (Fig. 3). Consistently with the established phylogenetic relationships, the percentage of cDNA sequences with hits in OlePic was proportional to the different species phylogenetic likelihood.

The annotation algorithm used, Sma3s, searches with three steps of significance that sequentially retrieve annotations from (i) already existing annotated sequences, (ii) orthologous sequences, and (iii) groups of sequences sharing a statistically significant pattern. Nevertheless, Sma3s retrieved annotations from Uniprot plant database for 48% of the transcriptome (75,772 unigenes).

All in all, ABySS reduced the original number of sequences by 99.99%. The percentage of GC was very similar to that of 42.5% reported previously for other olive assemblies.^30,41^ Unlike these recent studies, our starting material consisted exclusively of short reads. Nevertheless, the average length, integrity of assembled genes, and number of unigenes annotated were improved compared with the currently available, most complete olive transcriptome AS8.^30^ In addition, analysis of OlePic with FLN comparing with AS8 and *M. guttatus* (28,282 protein-coding transcripts) indicates a similar structure pattern in OlePic compared with AS8 (Supplementary Fig. S2). As expected, FLN found 27,207 *M. guttatus* transcripts with orthologue of which 18,344 were complete. FLN found 88,034 transcripts with orthologues in OlePic of which 11,436 were complete. This supports the contention that the use of short paired-end data can lead to an efficient assembly of transcriptomes, as previously anticipated.^42,43^ Furthermore, another mapping analysis with bowtie2 algorithm showed that 69% of AS8 sequences aligned locally in OlePic assembly. Taking into account that AS8 was constructed from different cultivars and more diverse tissues, global results indicate that our assembly is good enough to analyse gene-expression changes in olive tree; and, more importantly, it includes environmental-stress-response transcripts which are crucial for this study. The RNAseq analysis showed 6,309 unigenes differentially expressed with a minimum 8-fold change and 95% significance.

### 3.3. Short-term transient gene-expression changes in response to cold stress

**Figure 4.**
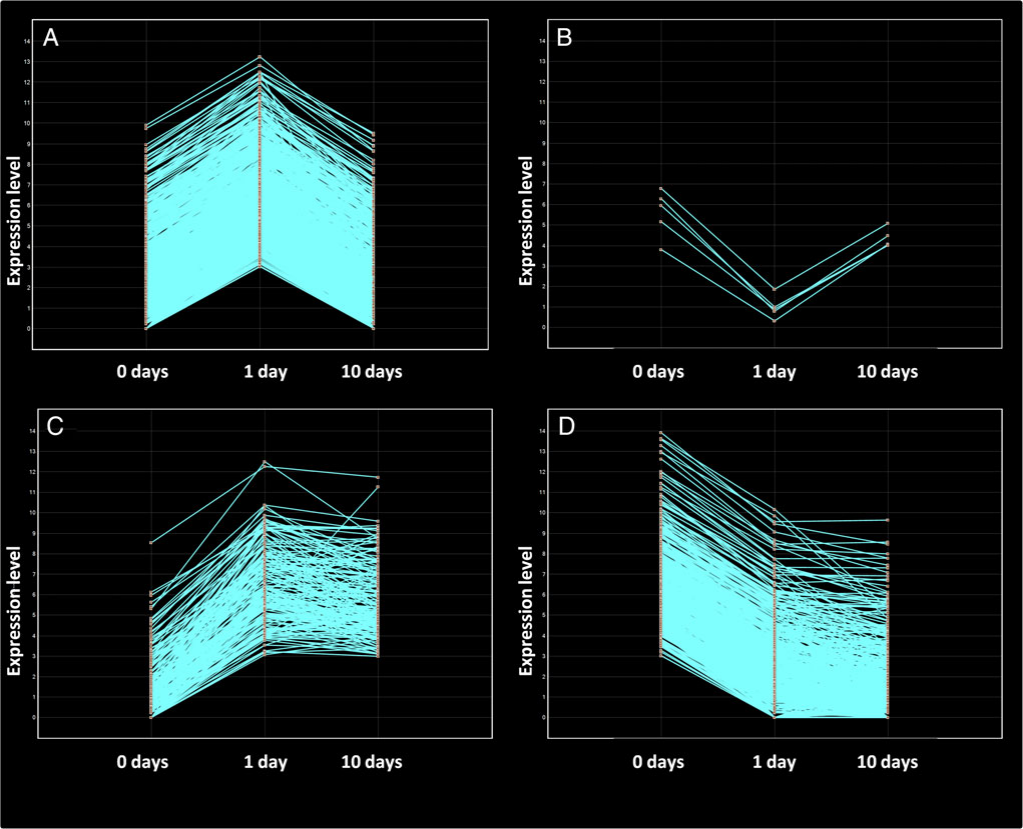
Unigenes with early expression changes in ‘Picual’ plants under prolonged low-temperature stress*.* Line graphs showing the expression over time of (A) the transient over-expressed genes, (B) the transient repressed genes, (C) the sustained over-expressed genes, and (D) the transient repressed genes.

The response of plants to prolonged stress stimuli may involve both transient reactions shortly decaying after the first moments of stress exposure and adaptive changes sustained over time. The result of comparing 0 and 1 day of cold exposure was that 1,694 unigenes were differentially expressed (8-fold change and 95% significance), 1,094 of them were over-expressed, and 600 were repressed after the first 24 h of low-temperature exposure. However, about half of these unigenes (896) returned to normal expression levels after 10 days of low-temperature exposure (890 over-expressed and only 6 repressed). These unigenes represent a group of genes showing a transient response to cold stress (Fig. 4A and B; Supplementary Fig. S3). This transient response resulted from the induction of expression for at least the first 24 h of cold stress in almost all genes. However, expression returned to normal levels after prolonged exposure to low temperature.

**Figure 5.**
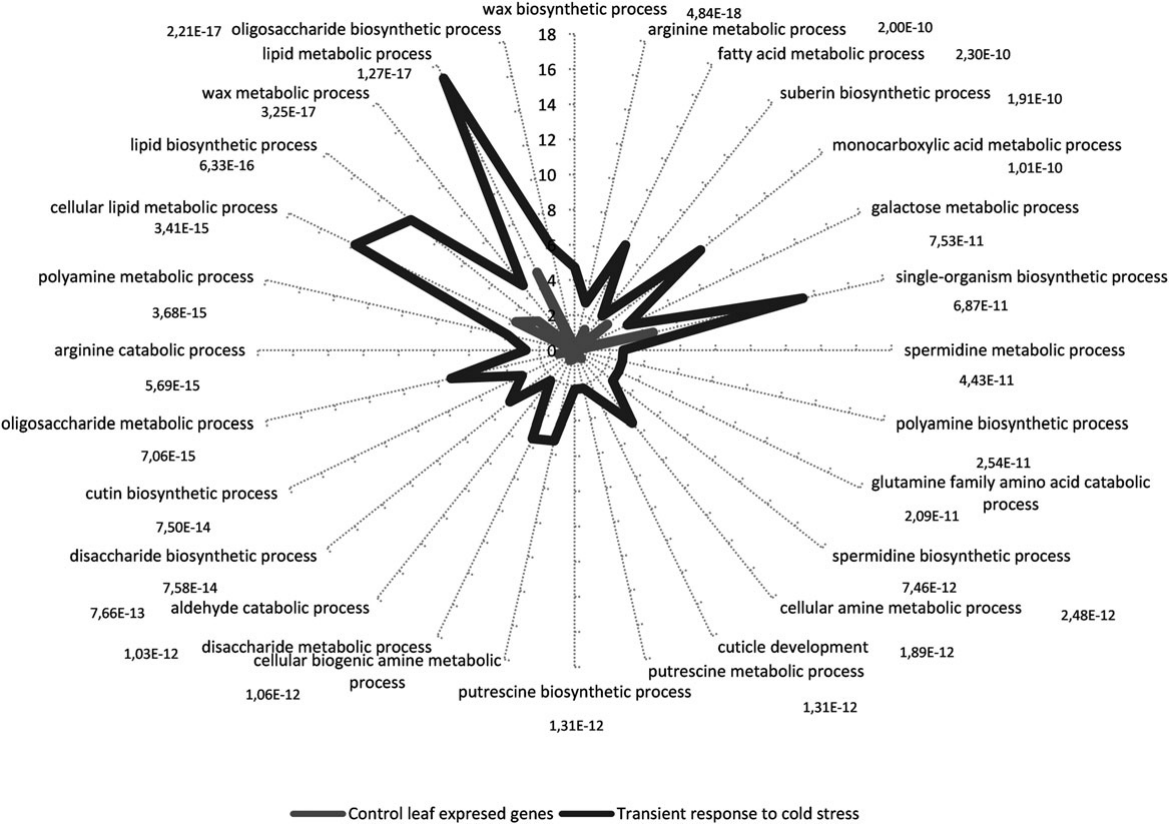
GO-term-enriched graph of biological process of transient response to cold stress*.* Up- and down-regulated unigenes, subset of annotated sequences were analysed. Node filter was set at FDR < 1e−7. Bars for early, short-term unigenes are labelled with their corresponding *P*-values in the Fisher's exact test against the expressed control leaf unigenes.

To assess the differences in functional classes between the transient-response group and the genes expressed in control conditions, we selected unigenes expressed in control leaves with a minimum of 2 RPKM as the reference group. The GO terms retrieved by Sma3s for 361 sequences from 910 transient unigenes and for 43,696 sequences from 97,532 unigenes expressed in control leaves were loaded in Blast2GO suite V2.7.0 to perform the statistical analysis of GO-term enrichment. Unchecked, the two-tail box was used to analyse only positive enrichment. The test was made choosing a filter cut-off value of FDR < 1e−7. The results are shown in Fig. 5 as the percentage of sequences which annotated for each ‘biological process' GO term for both control leaves and transient COR unigenes. Bars are labelled with their corresponding *P*-values in the Fisher exact test. Most of the GO terms were parent–child related and involved mainly in lipid polymers, oligosaccharide, and polyamine metabolism (Fig. 5).

Cutin and suberin are fatty-acid-derived polymers and are matrices for lipophilic cell-wall barriers that play different roles including protecting plants from abiotic stress. Unigenes that annotated for these processes were identified, including *GPAT5* (494,160 and 276,135), *FAE1* (unigenes 47,056, 481,778, and 497,978), and *CER1/WAX2* (unigenes 372,554, 24,079, 312,953, 306,126, 456,548, and 409,294). Here, we report six *CER*-like genes in olive, all of which respond transiently to cold stress. In addition, 81 unigenes annotated for seven GO terms associated with oligosaccharide metabolism (Fig. 5). Oligosaccharides are ubiquitous in plants and have been associated with freezing tolerance in various species. Four unigenes (362,593, 453,896, 511,359, and 174,049) were associated with the seven GO terms and coded for a stachyose synthase.

On the other hand, we observed a transient induction for several unigenes involved in polyamine biosynthesis including arginine decarboxylases (*ADC*) (unigenes 62,085, 57,082, 612,727, 22,384, 531,518, 266,717, 417,814, 417,815, 416,039, and 494,283), amine oxidase flavin domain-containing protein (unigenes 233,490 and 205,434), and peroxisome polyamine oxidase (unigenes 515,010 and 479,660).

Consequently, with most representative ‘biological processes' found, statistically significant GO terms for ‘biological function’ were arginine decarboxylase activity, aldehyde decarbonylase activity, oxidoreductase activity, carbon–carbon lyase activity, oxidoreductase activity, choline kinase activity, inositol 3-alpha-galactosyltransferase activity, and beta-amylase activity. No ‘biological component’ GO term was found for the chosen FDR cut-off.

### 3.4. Early long-term gene expression changes during cold acclimation

Together with the transient response to cold stress, a set of genes showing a sustained response to cold stress for a prolonged time period (10 days) was identified. Thus, 798 unigenes out of the 1,694 with significant 8-fold change between 0 and 1 day under low temperature still remained up-regulated 10 days under cold stress. Therefore, these genes are likely to take part in an adaptive response to low temperature. In this case, 204 unigenes were induced during the cold exposure, whereas 594 were repressed (Fig. 4C and D; Supplementary Fig. S3).

**Figure 6.**
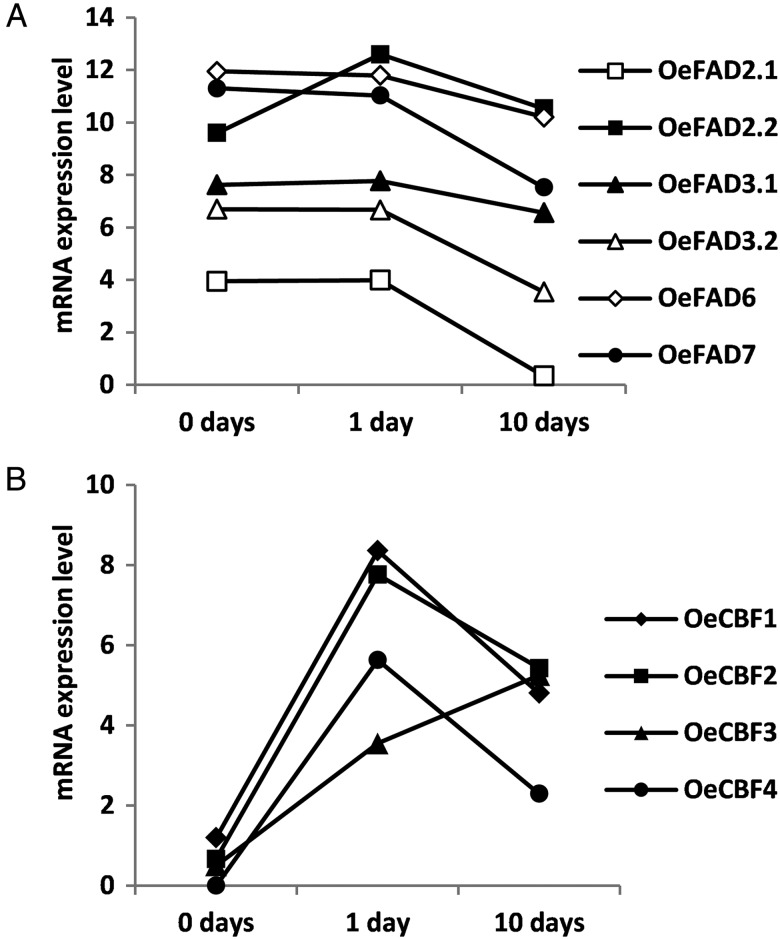
FAD and CBF expression in ‘Picual’ olive plants under prolonged low-temperature stress*.* Expression levels were obtained by RNAseq analysis with DNAStar Qseq software and are represented as log_2_ values. *X*-axis categories correspond to the time ‘Picual’ plants exposed to low temperature. (A) Represents the *OeFAD* gene expression pattern and (B) the *OeCBF* gene expression pattern.

**Figure 7.**
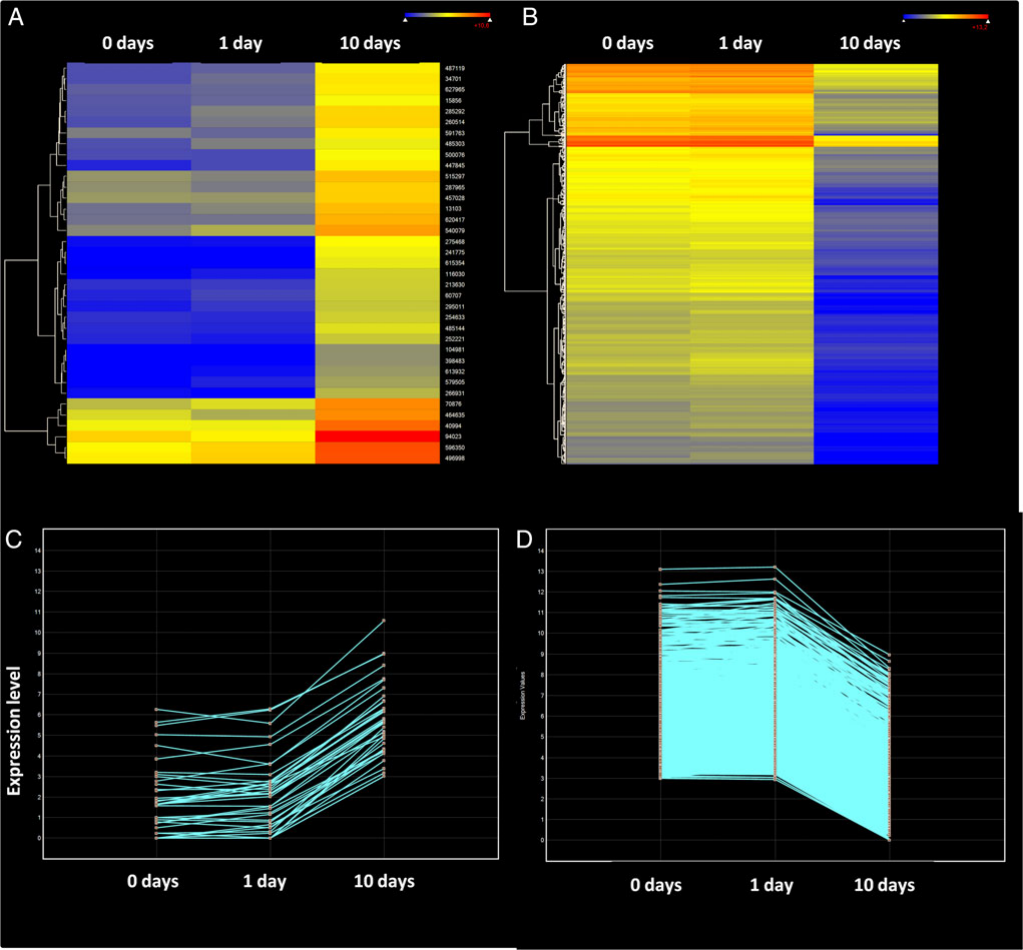
Unigenes with delayed expression changes in ‘Picual’ plants under prolonged low-temperature stress*.* Unigenes with 95% significant differential expression at 8-fold change when comparing both 0 and 1 day to 10-day cold-stressed plants. (A) Heat map of the induced genes after long time of low-temperature exposure. (B) Heat map of the repressed genes after a long time of low-temperature exposure. (C) Line graphs showing the expression over time of the delayed over-expressed genes. (D) Line graphs showing the expression over time of the delayed repressed genes.

One important adaptive response to cold is the induction of FADs to regulate the fluidity of the cellular membrane. Indeed, an increase in FAD activity relaxes cellular membrane rigidity.^44^ In olive, *FAD2.2* and *FAD7* increased their expression in epi–mesocarp cells during the oleogenic period.^17^ However, just *OeFAD2* transcription increased in both the embryo and the seed coat in cold-acclimated drupes.^18,45^ We have found in the olive transcriptome unigenes that the code for FAD2.1, FAD2.2, FAD6, FAD7, and two FAD3, one previously described that we have called FAD3.1 and a new one called FAD3.2. The RNAseq analysis has shown that *OeFAD2.2* is the only FAD-coding gene that increased its expression in response to cold in ‘Picual’ leaves (Fig. 6A). The *OeFAD2.2* was induced shortly after the start of the cold exposure and maintained a high expression level after 10 days of low-temperature treatment (Fig. 6A), even though a general tendency towards a reduction in transcription was observed for most genes (Fig. 7). This could probably be explained as a consequence of a diminished metabolism of the plant. This finding supports the idea that FAD2.2 is the desaturase involved in cold response in olive leaves, in agreement with previous results,^17,18,45^ suggesting that FAD2.2 is the main desaturase involved in cold response in olive.

Many *COR* genes are regulated by CBF transcription factors, which are up-regulated by the rise in the intracellular Ca^2+^ level as a consequence of falling temperatures.^16^ Hence, we searched for the presence of *CBF* orthologues in the olive transcriptome. We found four unigenes that were induced by cold in a sustained manner while the low temperature persisted. However, three of them decreased their expression between 1 and 10 days of cold exposure but still remained significantly higher than in control plants (Fig. 6B). The four *CBF* genes were strongly induced between 0 and 1 day of low-temperature exposure. Nevertheless, only *OeCBF3* showed a steady increase in its expression after 10 days of cold treatment, suggesting that *OeCBF3* might be responsible for inducing *COR* gene expression, showing a late response to cold stress (Table 1 and Fig. 6B). The sequence similarity between Arabidopsis CBF coding sequences and the *OeCBF* unigenes did not allow us to identify exact orthologues in the Arabidopsis *CBF* family. Thus, they were numbered without any correspondence to the Arabidopsis nomenclature (Table 1). This means that the family of *CBF* genes in olive consists of at least four genes with an unclear correspondence to the Arabidopsis annotation of *CBF1*, *CBF2*, or *CBF3*.

**Table 1.** *OeCBF* gene expression during prolonged low-temperature stress

Unigene	Name	0 day	1 day	10 days	Fold change 0 versus 1-day cold	Fold change 0 versus 10-day cold
389521	*OeCBF1*	1.19499	8.36177	4.80685	143.69	12.23
579079	*OeCBF2*	0.66667	7.76301	5.43121	136.84	27.18
52166	*OeCBF3*	0.5	3.54641	5.25041	8.26	26.92
247105	*OeCBF4*	0	5.63098	2.29248	49.56	4.9

Gene-expression level found by RNAseq analysis and expressed as log_2_ values.

**Figure 8.**
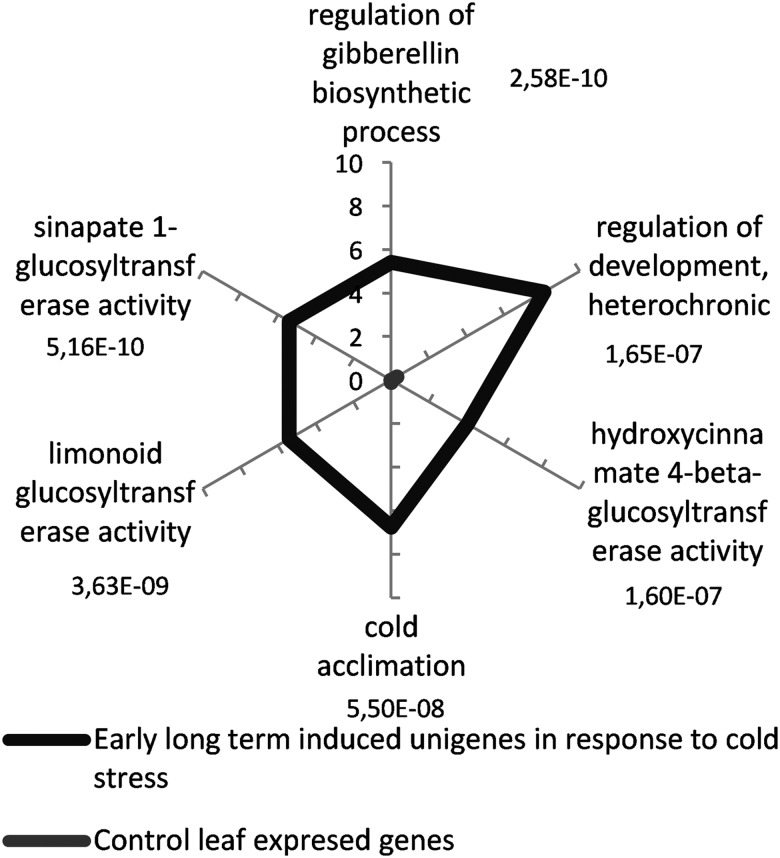
Biological process and function GO-term-enriched graph of early long-term-induced unigenes in response to cold stress. The subset of annotated sequences was analysed. The node filter was set at FDR < 1e−7. Bars for early long-term induced unigenes are labelled with their corresponding *P*-values in the Fisher's exact test against the expressed control leaf unigenes.

The GO-term enrichment analysis was performed as above with the GO terms retrieved by Sma3s for 74 sequences from 204 early long-term unigenes using a cut-off value of FDR < 0.001. The percentage of sequences that annotated for each ‘biological process' or ‘function’ GO term for both control and early long-term COR unigenes is shown in Fig. 8. No ‘biological component’ GO term was found for the chosen FDR cut-off. These unigenes were enriched in GO terms associated with three processes: regulation of gibberellin biosynthesis, cold acclimation, and development regulation. This result supports the idea that these genes are likely involved in an adaptive response to low temperature.

The GO-term enrichment analysis for 183 annotated from sequences of 594 early long-term down-regulated unigenes is plotted in Supplementary Fig. S4. Twenty unigenes were associated with nitrogen metabolism and 34 with photosynthesis (Supplementary Fig. S4). Therefore, our results indicate a down-regulation of photosynthesis and plant growth, and both ‘component’ and ‘function’ GO terms found were consistent with these results.

### 3.5. Late long-term gene-expression changes during cold acclimation

Plants respond to stress by global transcriptional changes that can be transient or sustained over time and with early- and late-responsive gene-expression alterations.^46^ Changes in gene expression in response to cold can be affected by differences in cold sensitivity. For instance, a general up-regulation trend in a chilling-tolerant genotype and an overall repression tendency in a sensitive genotype were found in rice.^47^ In our study, ‘Picual’ olive plants were subjected to low temperatures to which they can acclimate, and the cold-stress symptoms disappeared after 6 or 7 days of cold exposure (Fig. 1). We found a general trend towards gene repression after a period of 24 h at low temperature (Fig. 7). Thus, at 8-fold change and 95% significance, 4,551 unigenes were down-regulated between days 1 and 10 of low-temperature exposure and only 64 unigenes were over-expressed.

**Figure 9.**
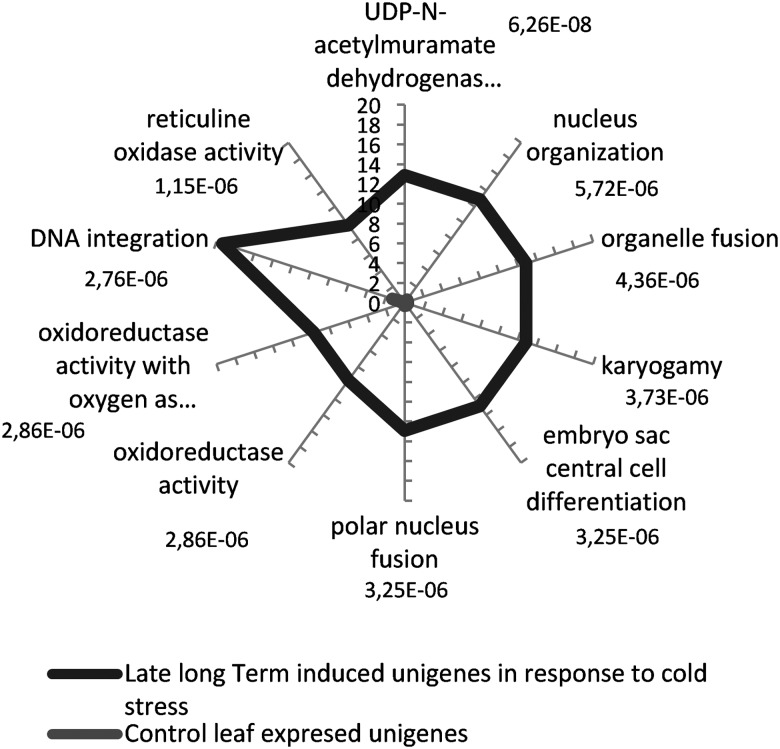
Biological process and function GO-term-enriched graph of late long-term-induced unigenes in response to cold stress*.* The subset of annotated sequences was analysed. The node filter was set at FDR < 0.01. Bars for late long-term-induced unigenes are labelled with their corresponding *P*-values in the Fisher's exact test against the expressed control leaf unigenes.

We performed the GO-term enrichment analysis as above with the GO terms retrieved by Sma3s for 31 sequences from 64 over-expressed late long-term unigenes using a cut-off value of FDR < 0.01. The percentage of sequences that annotated for each ‘biological process' or ‘function’ GO term for both control leaves and early long-term COR unigenes is shown in Fig. 9. No ‘biological component’ GO term was found for the chosen FDR cut-off. It was observed that the most representative processes were related to organelle fusion, nucleus organization, and DNA integration (Fig. 9). Among 20 sequences associated with organelle fusion and nucleus organization, four Flavin Adenin (FAD)-binding Berberine-like unigenes (unigenes 254,633, 435,666, 525,595, and 313,705) were shared by both processes. Six sequences were associated with DNA integration. They were annotated as Ty3-gypsy subclass, Gag-pol polyprotein, retroelement polyprotein-like, Copia-type polyprotein, and integrase (unigenes 104,981, 285,292, 627,965, 95,624, 510,781, and 36,107). Thus, they are putative transposable elements (TEs, jumping genes).

In summary, an extensive down-regulation of transcription in olive as a long-term response to cold was observed. The ‘biological processes’, ‘functions’, and ‘components’ associated with 2,147 from 4,551 repressed unigenes are shown in Supplementary Fig. S5. Results confirmed that unigenes related to photosynthesis and plant growth are inhibited mostly during a late response to cold. Further experimental evidence is required to determine whether AGO-like, bHLH-like, Berberine-like, and long terminal repeat retrotransposons are responsible for this major down-regulation during late cold acclimation in olive tree.

### 3.6. RNAseq validation by Q-RT–PCR

**Figure 10.**
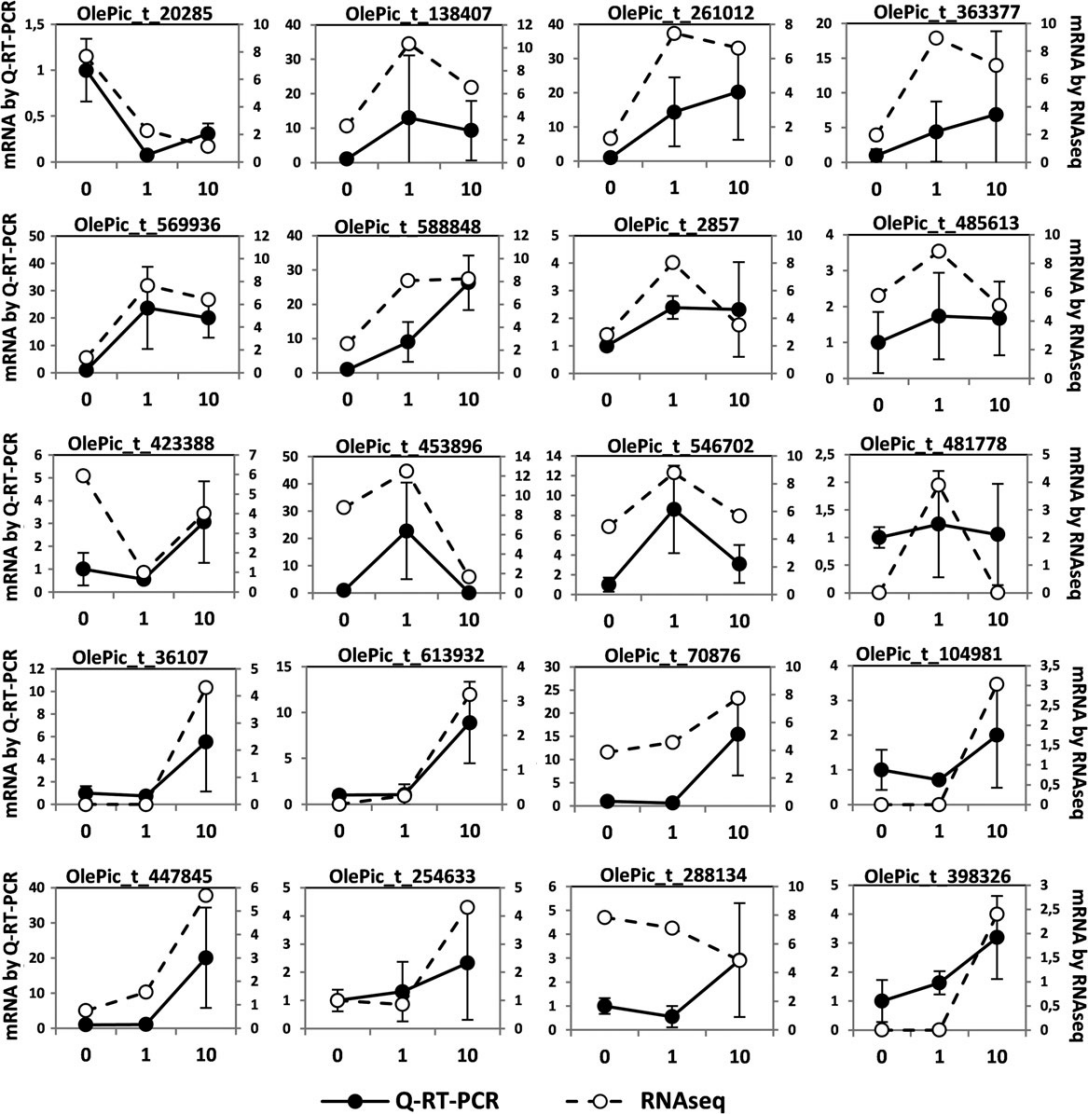
Analysis of mRNA level by Q-RT–PCR. The mRNA level of 20 randomly selected genes was analysed by Q-RT–PCR. The average of results obtained from three plants is represented (left *Y*-axis) together with the RNAseq data (right *Y*-axis).

The expression of 20 randomly selected genes was analysed by Q-RT–PCR in nine trees, three for each time of cold exposure. As a result, the pattern of expression observed was very similar between Q-RT–PCR and RNAseq results in 18 of the 20 genes analysed, while for OlePic_t_481778 the induction after 1 day of cold exposure was weaker by Q-RT–PCR than by RNAseq, and for OlePic_t_288134 the pattern was opposite at 10 days of cold exposure (Fig. 10). This result confirms that the expression patterns of mRNA obtained by RNAseq reflect real changes of gene expression.

### 3.7. Conclusions

Olive plants cv. Picual were sensitive to cold stress as observed by morphological and ROS enzymes changes. However, they were also able to fully recover after 5 days of low temperatures, indicating the existence of acclimation mechanisms to cold. A new transcriptome for stressed olive plants was built up and used to analyse the transcriptomic response to cold. We found 6,309 unigenes differentially expressed in response to cold, which were included in three types of responses leading to cold acclimation: a short-term transient response including 890 over-expressed and only 6 repressed unigenes, an early long-term response that consisted in 204 over-expressed and 594 repressed unigenes, and a late long-term response with 4,551 unigenes down-regulated between days 1 and 10 of low temperature exposure, and just 64 unigenes were over-expressed.

The results presented here suggest a complex acclimation response to cold in olive leaves with early transient or long-term and late transcriptional responses.

## Supplementary data

Supplementary data are available at www.dnaresearch.oxfordjournals.org.

## Funding

This work was supported by grant AGR-5948 from Junta de Andalucía (Consejería de Economía, Innovación y Ciencia) and Ministerio de Ciencia e Innovación. Funding to pay the Open Access publication charges for this article was provided by the University of Jaén.

## Supplementary Material

Supplementary Data
