# Supplementary material for: Early and delayed long-term transcriptional changes and short-term transient responses during cold acclimation in olive leaves
Source: DNA Res. 2014 Oct 16;22(1):1–11. doi: 10.1093/dnares/dsu033 (PMC4379972; doi:10.1093/dnares/dsu033)
Supplement: Supplementary Data [file supp_22_1_1__index.html]

Early and delayed long-term transcriptional changes and short-term transient responses during cold acclimation in olive leaves — Supplementary Data 

# Early and delayed long-term transcriptional changes and short-term transient responses during cold acclimation in olive leaves

## Supplementary Data

Supplementary Data

**Files in this Data Supplement:**

- Supplementary Figure 1 - tif file
- Supplementary Figure 2 - tif file
- Supplementary Figure 3 - tif file
- Supplementary Figure 4 - tif file
- Supplementary Figure 5 - tif file
- Supplementary Table 1 - doc file
- Supplementary Table 2 - doc file
