# Supplementary figures and images for: Early and delayed long-term transcriptional changes and short-term transient responses during cold acclimation in olive leaves
Source: DNA Res. 2014 Oct 16;22(1):1–11. doi: 10.1093/dnares/dsu033 (PMC4379972; doi:10.1093/dnares/dsu033)

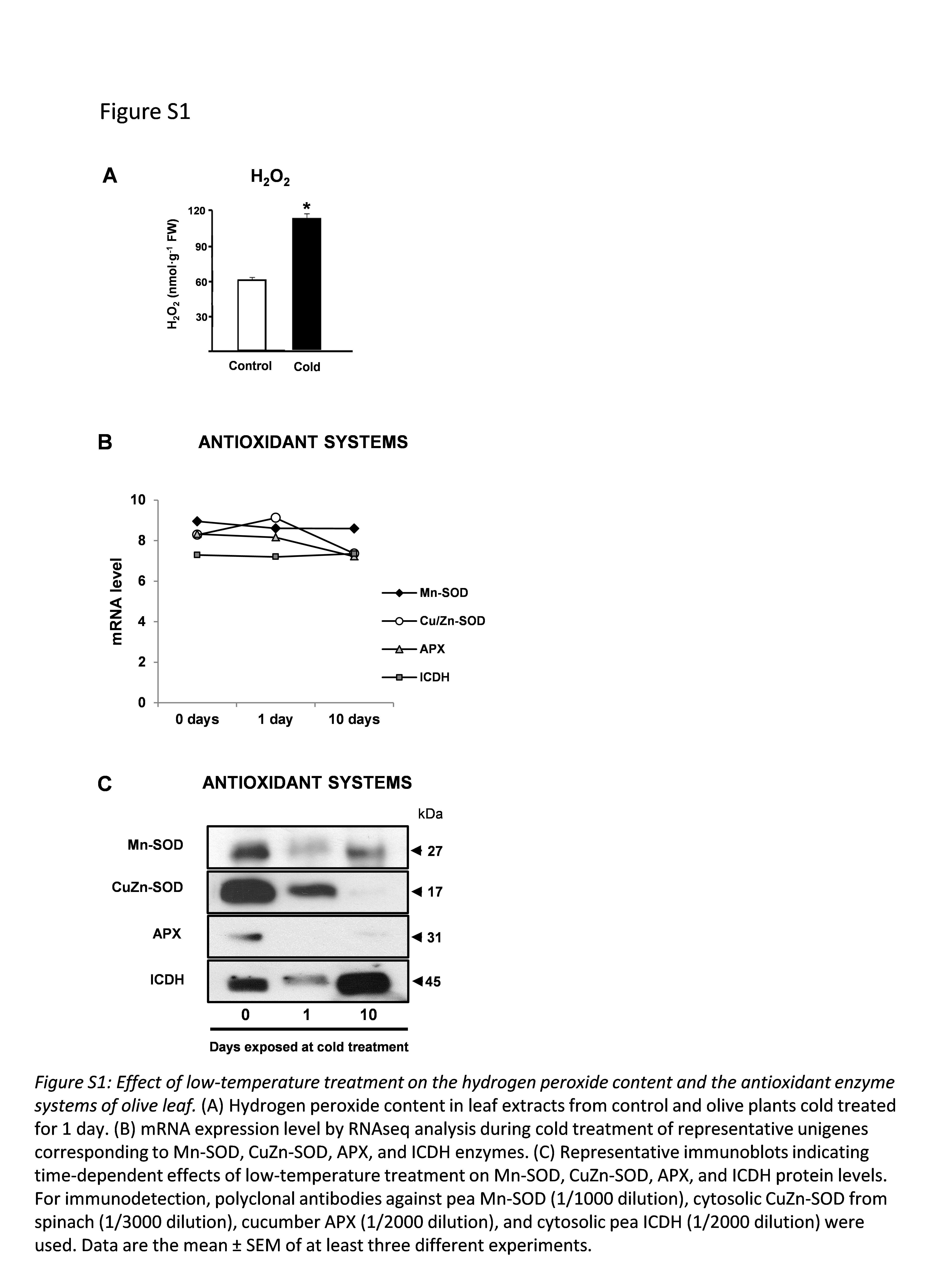

Supplement: Supplementary Data [file supp_dsu033_dsu033supp_fig1.tif]

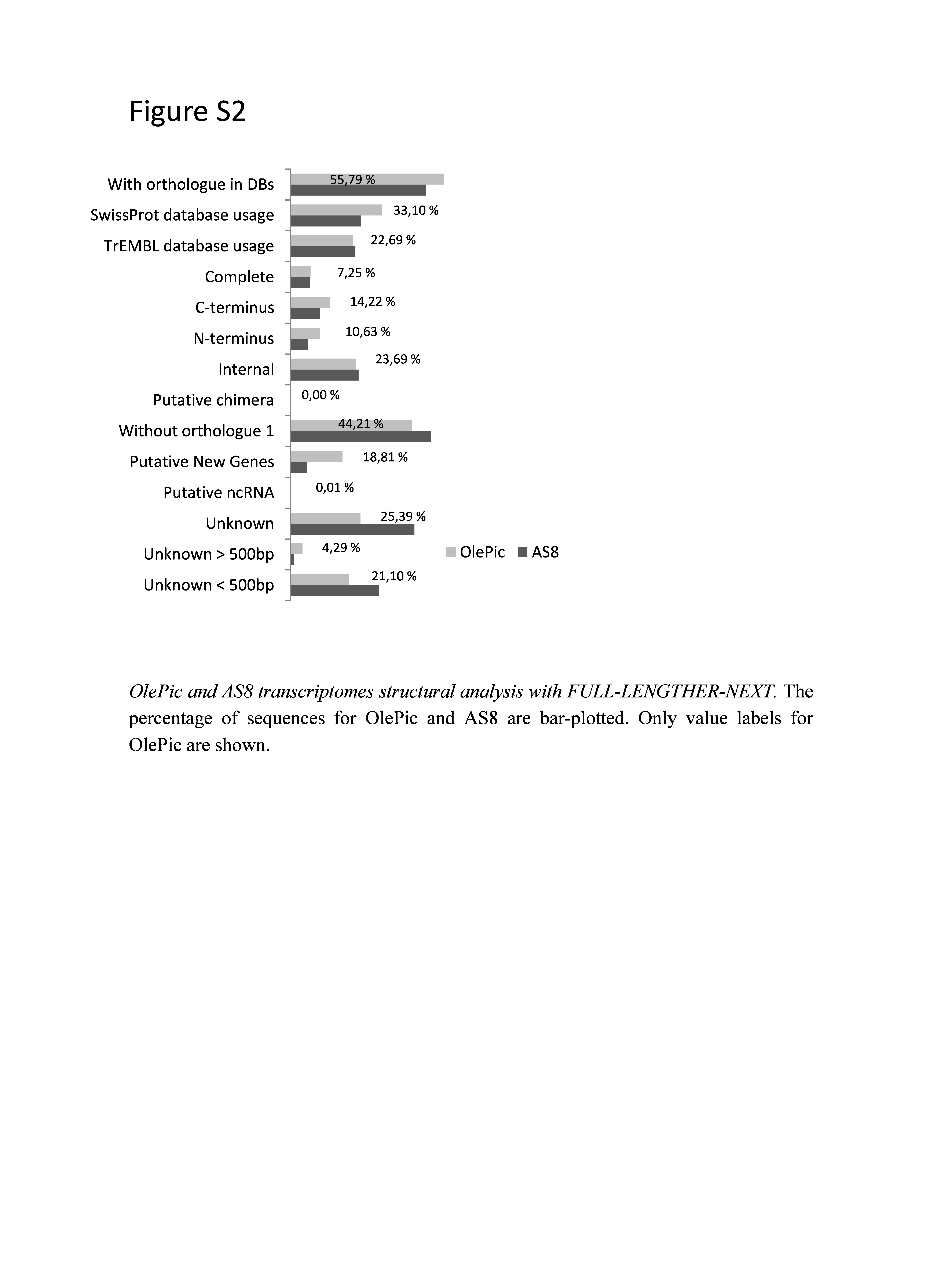

Supplement: Supplementary Data [file supp_dsu033_dsu033supp_fig2.tif]

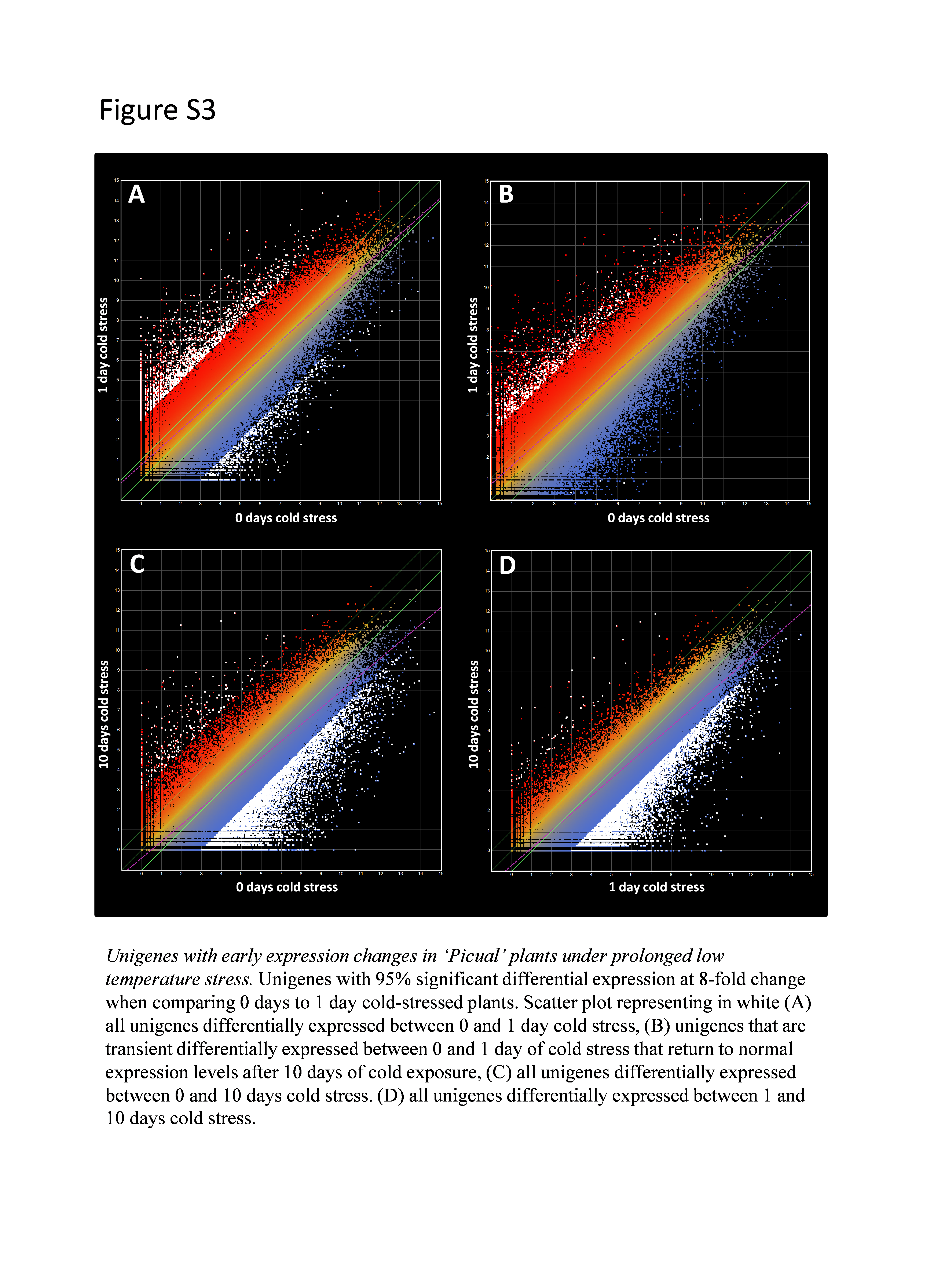

Supplement: Supplementary Data [file supp_dsu033_dsu033supp_fig3.tif]

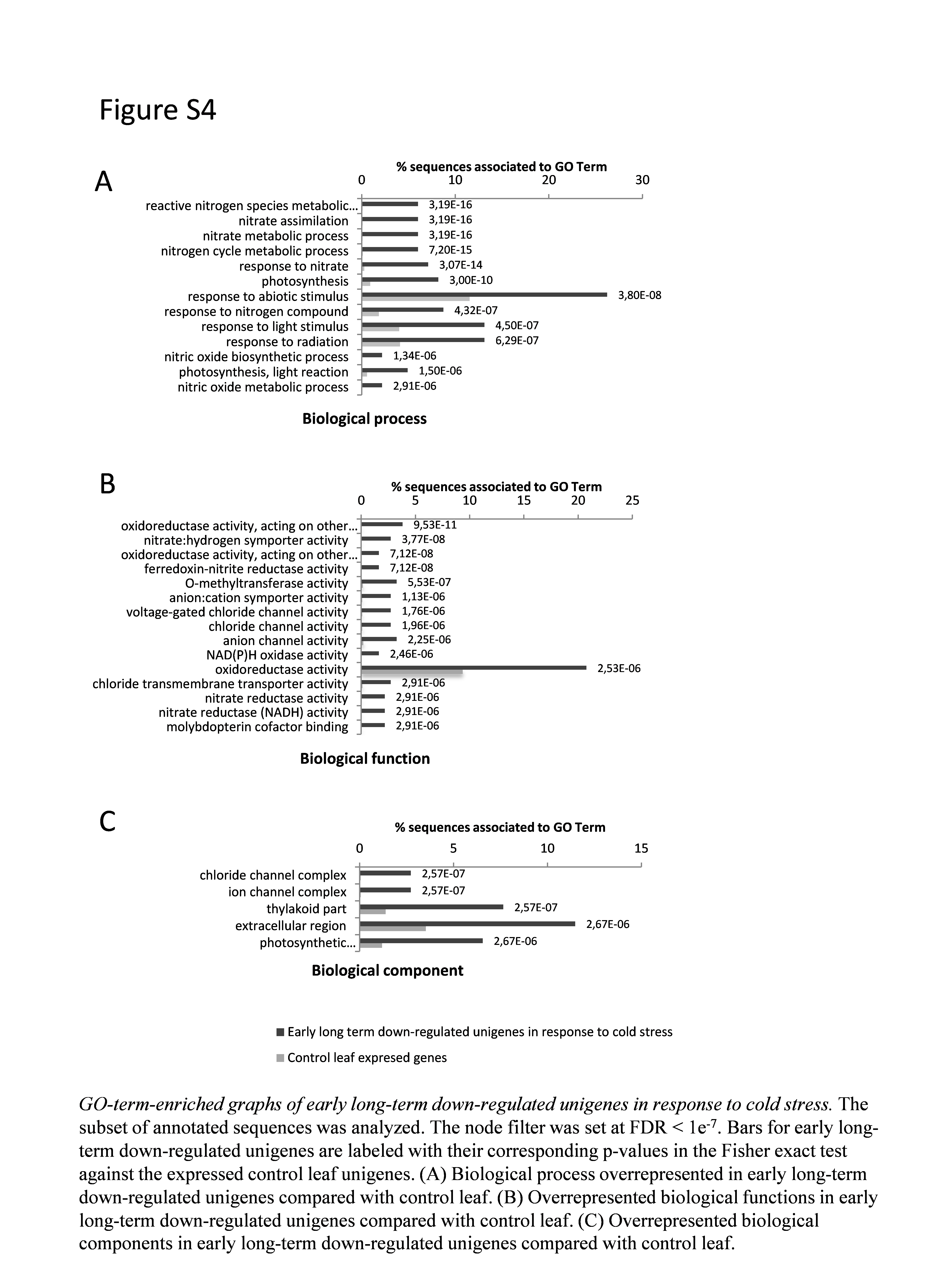

Supplement: Supplementary Data [file supp_dsu033_dsu033supp_fig4.tif]

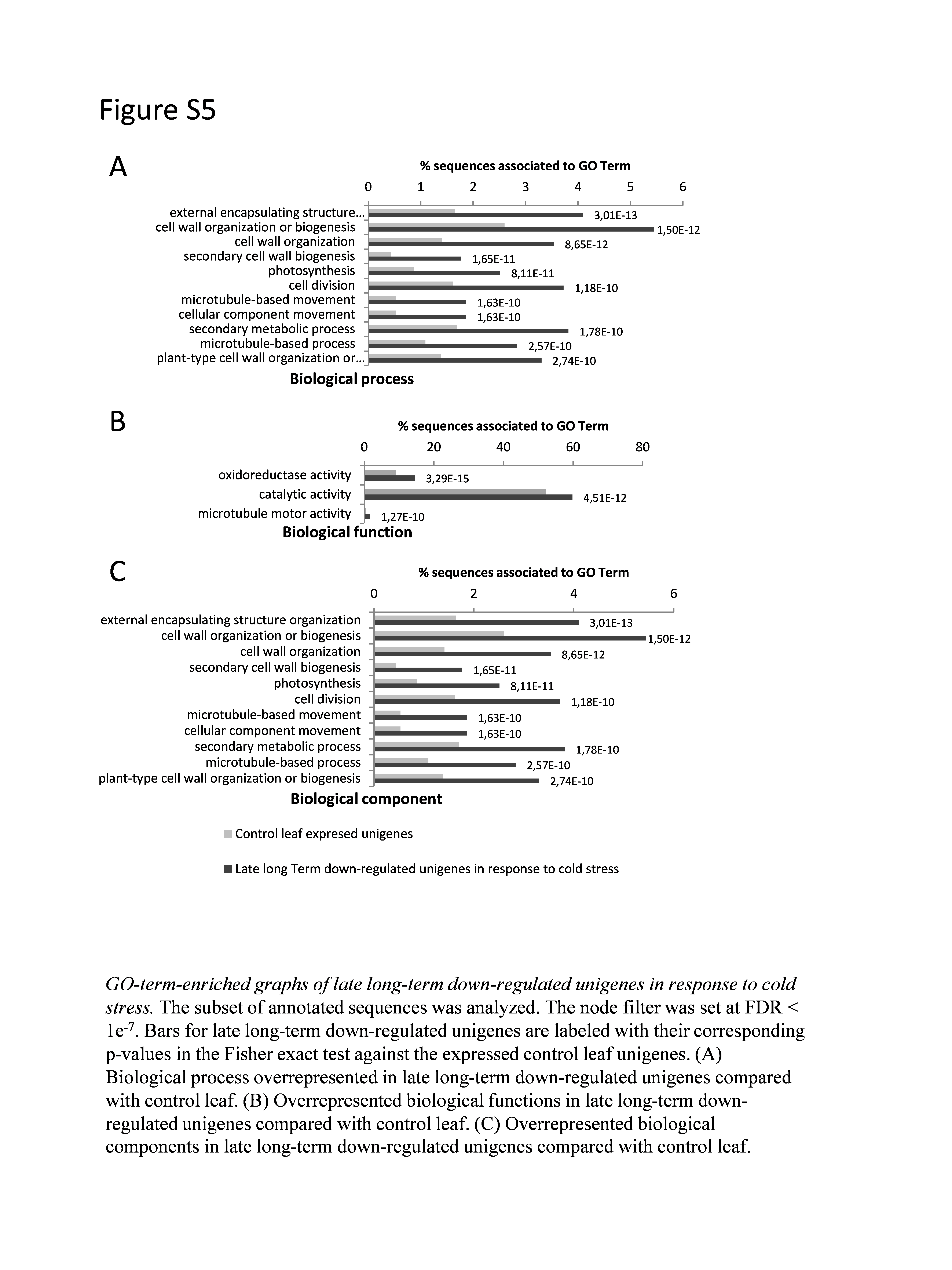

Supplement: Supplementary Data [file supp_dsu033_dsu033supp_fig5.tif]
