# Supplementary material for: Early and delayed long-term transcriptional changes and short-term transient responses during cold acclimation in olive leaves
Source: DNA Res. 2014 Oct 16;22(1):1–11. doi: 10.1093/dnares/dsu033 (PMC4379972; doi:10.1093/dnares/dsu033)
Supplement: Supplementary Data [file supp_dsu033_dsu033supp_table1.doc]

**Table S1:** *Primers for Q-RT-PCR.*

|  | **Primer name** | **Primer sequence** | **Amplicon size (bp)** |
| --- | --- | --- | --- |
| **OlePic_t_20285** | 20285F | TTATTTAAAGAAATCAAGTTTGAAGATGCTGAAGG | 160 |
|  | 20285R | AGCCCTACCATGGAGTTGAATCTGTCAC |  |
| **OlePic_t_138407** | 138407F | GAGAAAACGAGCTCAAGAATACAAAGAGCAG | 134 |
|  | 138407R | ACAATTGGAAAATTCAAGACTATACGCATTACG |  |
| **OlePic_t_261012** | 261012F | CATCTTCAGCCAGCTTCCACAAAACC | 181 |
|  | 261012R | AGAGATCCCAGCATTGGCAGTGACTC |  |
| **OlePic_t_363377** | 363377F | TCCTCAGAAGTTCTACGATATCTCGTGTTCC | 142 |
|  | 363377R | GCAAGCATTAGGCTGAAATATTTGTATGTGG |  |
| **OlePic_t_569936** | 569936F | GGATCATTTGTTCGCGTCTACACCAGTG | 137 |
|  | 569936R | GAATAGGCATCCCCACATGATTCTCTCTG |  |
| **OlePic_t_588848** | 588848F | TGAGTTATAGGGTTGGAGGTCCTATCAATGG | 173 |
|  | 588848R | AGGCTACCAAGCTTTACTGTATGATTAGCAAGC |  |
| **OlePic_t_2857** | 2857R | CAGATTCACTAAACAAATCCCAGCAACTCTC | 143 |
|  | 2857F | GATAGTGGAGATTGAAAAGGCAAAAGAAGC |  |
| **OlePic_t_485613** | 485613F | CTGCCATCTAAGAAGCCAATTACAAGAAGC | 186 |
|  | 485613R | GTAAAACTGGATATGGTCGGGTCACAGAG |  |
| **OlePic_t_423388** | 423388F | AAAAATGATTATACTCCCTGAGTGATCGTTGC | 124 |
|  | 423388R | CCCATGCTATATTTATCCCAAAGCTTGTTACC |  |
| **OlePic_t_453896** | 453896F | CATACTCTAGAGTGTGTTTGTGAGGACCATGG | 159 |
|  | 453896R | CTTTGTAGCGAGAAAGAAGAAATCGTTGC |  |
| **OlePic_t_546702** | 546702F | TGTTTATATGGAAAAATAATGGCTAGGGTTGC | 112 |
|  | 546702R | CTTTAAACTCGAGAATTCAAGATCCTTTCAGC |  |
| **OlePic_t_481778** | 481778F | CTAGACTAATCTTAAGCAGCGAGCCTAAAAGC | 192 |
|  | 481778R | TCTTCTCGAAAAGAGATTCCATTGCTGAG |  |
| **OlePic_t_36107** | 36107F | TCTTTCTCTCAGCTACTCCATTTTGTTGTGG | 154 |
|  | 36107R | AAGACAAAGTGGATTTCTAATAAAGACGTTGAGG |  |
| **OlePic_t_613932** | 613932F | ATTGGGGTACTTGTGCGAGAATGTGC | 117 |
|  | 613932R | CTTTGTGCATGATTCTTGAATTTTGTCTCTG |  |
| **OlePic_t_70876** | 70876F | GCAGGAGCTCTCCACCACTTGTACTTCC | 179 |
|  | 70876R | CTTCACTTCAAATCACCAAACAATCAATGC |  |
| **OlePic_t_104981** | 104981F | CACTTATCAGTTTTAATCCGCTAACACCTATGG | 117 |
|  | 104981R | CCTTTTCATGGAGTGTTTTCACAATCTCG |  |
| **OlePic_t_447845** | 447845F | GAAATCAAATTCAAACCCAAGGAAGCAG | 112 |
|  | 447845R | CAAGCAAAAAATGAATATCAAGAAGAAGAAGAGG |  |
| **OlePic_t_254633** | 254633F | GGATCTGTTCATCAGAGTCATCATTACAAAAGC | 129 |
|  | 254633R | CTTTCGTCCATTATTCCAAGCAATCTATCG |  |
| **OlePic_t_288134** | 288134F | ACGACACCGACTCTAGGCGTATGTGG | 159 |
|  | 288134R | TTTGGAAGAGCGTGAAGGTATAGTTGACG |  |
| **OlePic_t_** | 398326F | GTCGCACTGATAAACATTGTGTGCTACTACG | 114 |
|  | 398326R | ACCAGCTAACATCCCTGACCAGATTCC |  |
