# Supplementary material for: Early and delayed long-term transcriptional changes and short-term transient responses during cold acclimation in olive leaves
Source: DNA Res. 2014 Oct 16;22(1):1–11. doi: 10.1093/dnares/dsu033 (PMC4379972; doi:10.1093/dnares/dsu033)
Supplement: Supplementary Data [file supp_dsu033_dsu033supp_table2.doc]

Table S2: Transcriptome assembly scheme and data.

| **Samples** | **Biosample accession number** | **SRA accession number** | **no of reads** | **Average Quality scores (Phred)** | **no of scaffolds after**  **ABySS-paired end assembly (Average length in bp)** | **no of contigs after**  **ABySS-single end re-assembly**  **(Average length in bp)** |
| --- | --- | --- | --- | --- | --- | --- |
| Unstressed plant roots | SAMN02937426 | SRR1525051 | 18,974,562 | 35,12 | 134,945 (412) | 174,965 (544) |
|  |  | SRR1525052 | 19,096,074 | 35,15 | 135,037 (414) |  |
| Roots wounding 8h | SAMN02937427 | SRR1525231 | 45,857,680 | 34,83 | 177,380 (334) |  |
|  |  | SRR1525237 | 45,753,537 | 34,85 | 175,661 (336) |  |
| Roots wounding 24h | SAMN02937428 | SRR1524947 | 26,775,754 | 34,93 | 276,359 (267) |  |
|  |  | SRR1524948 | 26,585,586 | 34,96 | 255,400 (274) |  |
| Roots wounding 48h | SAMN02937429 | SRR1524949 | 22,547,755 | 35,05 | 165,626 (343) |  |
|  |  | SRR1524950 | 22,620,697 | 35,09 | 165,481 (345) |  |
| Roots wounding 7 days | SAMN02937430 | SRR1524951 | 30,930,898 | 35,46 | 128,890 (401) |  |
|  |  | SRR1524952 | 31,030,239 | 35,50 | 132,426 (398) |  |
| Roots infected by *Verticillium dahliae* 48h | SAMN02937431 | SRR1525086 | 27,214,837 | 35,41 | 203,581 (241) |  |
|  |  | SRR1525087 | 27,143,904 | 35,45 | 273,413 (234) |  |
| Roots infected by *V. dahliae* 7 days | SAMN02937432 | SRR1525113 | 27,681,432 | 34,91 | 151,692 (302) |  |
|  |  | SRR1525114 | 27,390,675 | 34,85 | 153,140 (302) |  |
| Roots infected by *V. dahliae* 15 days | SAMN02937433 | SRR1525213 | 22,286,016 | 34,63 | 167,987 (290) |  |
|  |  | SRR1525114 | 22,030,842 | 34,56 | 167,059 (289) |  |
| Unstressed plant leaves | SAMN02937434 | SRR1525224 | 28,852,727 | 35,55 | 224,702 (298) |  |
|  |  | SRR1525226 | 28,899,046 | 35,58 | 232,393 (290) |  |
| Cold-stressed leaves 24h | SAMN02937435 | SRR1525284 | 35,059,159 | 35,24 | 174,038 (329) |  |
|  |  | SRR1525285 | 34,622,386 | 35,19 | 277,192 (267) |  |
| Cold-stressed leaves 10 days | SAMN02937436 | SRR1525286 | 11,131,408 | 35,59 | 96,268 (413) |  |
|  |  | SRR1525287 | 11,074,162 | 35,63 | 97,524 (408) |  |
| Leaves of plants with wounded roots 15 days | SAMN02937437 | SRR1525415 | 47,498,270 | 35,62 | 188,406 (286) |  |
|  |  | SRR1525416 | 26,325,267 | 35,89 | 185,239 (302) |  |
| Leaves of plants infected by *V. dahliae* 15 days | SAMN02937438 | SRR1525436 | 37,391,858 | 35,56 | 242,255 (308) |  |
|  |  | SRR1525437 | 36,899,984 | 35,52 | 216,101 (309) |  |
